# Differential induction of host cell autophagy by virulent and precocious strains of *Eimeria tenella* *in vitro* and *in vivo*

**DOI:** 10.14202/vetworld.2026.180-190

**Published:** 2026-01-20

**Authors:** Li Zhang, Ying-ying Chen, Hong-hui Zhang, Xiao-zhen Cui, Ming-xue Zheng, Long-long Zheng

**Affiliations:** Veterinary Pathology Laboratory, College of Veterinary Medicine, Shanxi Agricultural University, Taigu Shanxi, 030801, China

**Keywords:** autophagy, coccidiosis, *Eimeria tenella*, host–parasite interaction, live attenuated vaccine, poultry disease, precocious strain, virulent strain

## Abstract

**Background and Aim:**

*Eimeria tenella* is the most pathogenic species affecting chickens and a leading cause of economic loss due to coccidiosis. While live vaccines using virulent or attenuated strains are effective, they can still cause intestinal damage and reduce weight gain. Autophagy, a crucial host cell response during intracellular parasitic infections, shows variations in induction between virulent and precocious *E. tenella* strains that are not yet well understood. This study compares how host cell autophagy is triggered by the virulent *E. tenella* Shanxi strain (Tsx) and precocious Tsx (PTsx) strains, both *in vitro* and live animal experiments.

**Materials and Methods:**

Primary chick embryo cecal epithelial cells and specific pathogen-free chickens were infected with either low or high doses of Tsx or PTsx. Infection rates were determined through hematoxylin and eosin (H&E) staining. Autophagy levels were assessed by quantifying *Beclin-1* mRNA expression via reverse transcription quantitative real-time polymerase chain reaction, evaluating LC3II puncta accumulation through immunofluorescence (IF), and calculating LC3II/I ratios using Western blot. *In vitro* experiments were carried out from 4 to 120 h post-infection, whereas *in vivo* evaluations took place on day 5 after inoculation.

**Results:**

*In vitro*, infection rates did not differ significantly between Tsx and PTsx groups during early stages (4–72 h), but Tsx showed significantly higher infection rates at 120 h. Both strains induced autophagy in a dose-dependent manner, as evidenced by increased *Beclin-1* mRNA expression, LC3II puncta, and LC3II/I ratios compared with controls. These autophagy markers were consistently higher in Tsx-infected cells than in PTsx-infected cells at equivalent doses. *In vivo* findings mirrored *in vitro* trends, with stronger autophagy activation observed in Tsx-infected chickens, particularly at high doses. Autophagy activation was markedly amplified *in vivo* compared with *in vitro*, indicating the influence of the intestinal microenvironment.

**Conclusion:**

The highly virulent *E. tenella* strain Tsx causes intense and prolonged autophagy in host cells, while the less aggressive PTsx strain triggers a milder autophagic response. The level of autophagy activation is directly related to the parasite’s virulence and infection dose. These results show that excessive autophagy plays a significant role in intestinal damage during *E. tenella* infection and highlight that reducing host autophagy activation is crucial for developing more effective live attenuated coccidiosis vaccines.

## INTRODUCTION

Chicken coccidiosis is a serious intracellular protozoan disease caused by *Eimeria* species infecting chickens’ intestines. Globally, it leads to economic losses of up to £10.4 billion annually due to reduced productivity, higher mortality, and costs for prevention and treatment [1]. Among thes*e* species, *Eimeria tenella* is the most pathogenic and causes the greatest economic impact in poultry farming [2-4]. Due to concerns over drug residues and resistance to anticoccidials, immunization with live virulent or attenuated *E. tenella* vaccines has become a key strategy for controlling coccidiosis [5]. Studies have shown that virulent *E. tenella* Shanxi strain (Tsx) elicits strong immune responses but also damages the cecal epithelial cells and intestinal mucosa through apoptosis regulation [6, 7]. Conversely, precocious strains, which are less pathogenic but still immunogenic, are preferred for live vaccines. Precocious Tsx (PTsx), derived from Tsx after 15 passages, exhibits significantly reduced virulence and is widely used in vaccine research [8, 9]. However, vaccination with precocious strains can slightly restrict weight gain and cause minor intestinal damage in chickens [8, 10, 11]. Therefore, understanding how these strains impact the host, especially the mechanisms of PTsx-induced damage and ways to reduce vaccine-related growth suppression, remains a crucial area for further research.

Autophagy is a unique form of programmed cell death, distinct from apoptosis and necrosis. It serves as both a fundamental physiological process and a defense mechanism against environmental stress, often referred to as type II programmed cell death [12, 13]. *In vitro* studies show that infection with the virulent *E. tenella* strain Tsx activates autophagy in chick embryo cecal epithelial cells [14]. However, the pathogenic effects of *E. tenella* are intricately regulated by the “host cell–immune system–gut microbiota” axis. Standard *in vitro* culture systems only mimic cell-level host–parasite interactions and cannot fully replicate the complex intercellular regulatory networks observed *in vivo*. Notably, the virulent Tsx strain and its attenuated derivative PTsx vary significantly in virulence, biological traits, and the extent of intestinal inflammation and mucosal damage they cause, highlighting the importance of combining *in vitro* and *in vivo* studies to better understand strain-specific host responses.

Although live virulent and precocious *E. tenella* strains are widely used to control chicken coccidiosis, the cellular mechanisms behind strain-specific pathogenicity and vaccine side effects are still not fully understood. Studies have shown that virulent strains can trigger host cell autophagy and apoptosis, but most research either focuses solely on virulent strains or uses limited *in vitro* models. There is a lack of direct, systematic comparisons between virulent and attenuated (precocious) strains regarding autophagy induction, especially across both *in vitro* and *in vivo* infection settings. Additionally, while precocious strains like PTsx are generally considered safer as vaccines, vaccinated chickens often show mild growth delays and intestinal changes, indicating unresolved host stress responses. It remains unclear how much host cell autophagy contributes to these effects and whether autophagy activation varies in magnitude and nature between the strains in a dose-dependent way. Furthermore, traditional *in vitro* models do not account for the impact of the intestinal microenvironment, immune factors, and microbiota on autophagy regulation, creating a significant gap in understanding how cellular results translate to real-world outcomes.

The present study aimed to systematically characterize and compare host cell autophagy responses induced by the virulent *E. tenella* strain Tsx and its precocious attenuated derivative PTsx under both *in vitro* and *in vivo* conditions. Specifically, this study sought to (i) evaluate the effects of strain virulence and infection dose on host cell autophagy by assessing key autophagy markers, including *Beclin-1* expression and LC3II accumulation, (ii) compare temporal autophagy dynamics between primary chick embryo cecal epithelial cells and chicken cecal tissues, and (iii) elucidate the relationship between autophagy activation, parasite virulence, and intestinal injury. By addressing these objectives, this work aims to provide mechanistic insights into *E. tenella*–host interactions and to establish a theoretical basis for optimizing the safety and efficacy of live attenuated coccidiosis vaccines.

## MATERIALS AND METHODS

### Experimental animals and ethical approval

This study was approved by the Animal Experiment Committee of Shanxi Agricultural University (ethics approval number: SXAU-EAW-2022CE.GH.003007156). A total of fifty 14-day-old specific pathogen-free (SPF) White Leghorn chicks (mixed sex, balanced across groups) were obtained from Beijing Meri Avigon Laboratory Animal Technology Co., Ltd. (Beijing, China) and reared under strictly controlled pathogen-free conditions.

### Parasites and preparation of sporozoites

The virulent *E. tenella* strain (SX010323, Tsx) and the attenuated precocious strain (SX010323P15, PTsx) were obtained from the Laboratory of Veterinary Pathology at the College of Veterinary Medicine, Shanxi Agricultural University in Shanxi, China. The Tsx strain was originally isolated in 2008 from the cecal tissues of broiler chickens showing clinical coccidiosis, characterized by bloody diarrhea and severe cecal lesions, on a commercial poultry farm in Shanxi Province. It had a prepatent period of 141 hours. The PTsx strain was derived from Tsx after 15 successive passages involving precocious development and maintained a stable prepatent period of 120 h. Oocysts were incubated in 2.5% potassium dichromate at 28°C for 3 days. After grinding the oocysts, sporozoites were released and incubated with 0.25% trypsin and 0.5% bile salt at 37°C for 2 h, then washed three times with phosphate-buffered saline (PBS, pH 7.4). Sporozoite viability was checked by blood cell counting and trypan blue exclusion, with activity ≥90%. Excystation of Tsx and PTsx sporozoites was performed as described previously [15].

### Primary culture of chick embryo cecal epithelial cells

Chick embryo cecal epithelial cells (2 × 10^5^ per well, in 6-well plates, with 100 U/mL penicillin and 100 μg/mL streptomycin) were isolated from sixty 15-day-old SPF chick embryos (Merial Vital Corp., Beijing, China) following established protocols [16]. These cells were cultured in Dulbecco’s modified Eagle’s medium medium supplemented with 2.5% fetal bovine serum and 0.1 μg/mL epidermal growth factor at 41°C in 8% CO_2_.

### Experimental protocol *in vitro*

When cell adherence reached approximately 90%, chick embryo cecal epithelial cells were randomly divided into five experimental groups. The control group was not infected with *E. tenella*. Treatment groups were exposed to PTsx or Tsx sporozoites at low (2.0 × 10^5^ per well) or high (4.0 × 10^5^ per well) doses. Cells were collected at 4, 24, 72, and 120 hours after infection. Each experiment was independently repeated five times, with culture media refreshed every 48 hours. Infected cells were identified using H&E staining. For each sample, 500 cells were counted in 10 randomly chosen fields at ×400 magnification by a blinded observer. The infection rate was calculated as: (number of infected cells/total cells counted) × 100%.

### Experimental protocol *in vivo*

Fifty 14-day-old SPF chicks with comparable body weights were randomly divided into five groups. The blank control group was not inoculated with *E. tenella*. Experimental groups received sporulated oocysts (1 mL) of PTsx or Tsx by oral gavage at low (4,500 oocysts/chicken) or high (45,000 oocysts/chicken) doses. Chickens were maintained at 30°C ± 1°C with 55% ± 5% relative humidity under a 12 h light/12 h dark photoperiod and had ad libitum access to anticoccidial-free commercial feed (crude protein ≥18%) and water.

### IF detection of LC3II accumulation *in vitro*

Cell monolayers were fixed with 4% paraformaldehyde at 25°C for 20 min, permeabilized with 0.4% Triton X-100 for 5 min, and blocked with 5% goat serum for 60 min. The cells were then incubated overnight at 4°C with anti-LC3B rabbit antibody (Sigma-Aldrich; lot: 046M4787V) diluted 1:2000, followed by five PBS washes. Subsequently, they were incubated at 37°C for 1 h with Fluorescein isothiocyanate-conjugated goat anti-rabbit IgG secondary antibody (Bioss, Beijing, China) diluted 1:500. After washing five times with PBS, nuclei were stained with Hoechst 33342 (Beyotime, Shanghai, China) for 10 min. The samples were mounted with an anti-fluorescence quenching agent and analyzed under a fluorescence microscope (Olympus BX53, Japan). Nuclei showed blue fluorescence, while autophagosomes appeared as green puncta [17]. LC3II puncta were quantified by counting 100 cells per group using ImageJ software.

### Quantification of *Beclin-1* mRNA expression *in vitro* and *in vivo*

Total RNA was isolated from chick embryo cecal epithelial cells at 4, 24, 72, and 120 hours post-infection with *E. tenella*, as well as from chicken cecal tissues on day 5 after inoculation, using RNAiso Plus (Takara, Code No. 9109, lot: AHF1822A). The RNA samples underwent DNase treatment and were reverse-transcribed into cDNA with the PrimeScript™ RT reagent kit (Takara, Code No. RR047A, lot: AHF1207A). The concentration and purity of the cDNA were determined using a μDrop Duo Ultra microplate reader and a Multiskan SkyHigh spectro-photometer (Thermo Scientific™, USA). Quantitative real-time polymerase chain reaction (PCR) was carried out using TB Green® Premix Ex Taq™ GC (Takara, Code No. RR071A, lot: AHF1068A) on a CFX Connect™ Real-Time PCR Detection System (Bio-Rad). The primers for *Beclin-1* were newly designed (F: 5′-TGGCTTTCTT GGACTGTGTG-3′; R: 5′-ACCACCACTGCCACCTGTAT-3′; reference sequence: NM-001006332.1). *β-actin* was used as the internal control (reference sequence: L08165.1; F: 5′-CACCACAGCCGAGAGAGAAAT-3′; R: 5′-TGACCATCA GGGAGTTCATA GC-3′). The amplification protocol included an initial denaturation at 95°C for 30 seconds, followed by 40 cycles of 95°C for 15 seconds and 59°C for 34 seconds. Relative gene expression levels were calculated using the 2^ΔΔCt^ method.

### Western blot analysis of LC3 expression *in vitro* and *in vivo*

Total protein was extracted from chick embryo cecal epithelial cells and chicken cecal tissues using RIPA lysis buffer (Beyotime Biotechnology; lot: 051018180521). Equal amounts of protein (20 μg per sample) were separated on 12% sodium dodecyl sulfate polyacrylamide gel electrophoresis gels under reducing conditions, transferred to nitrocellulose membranes via tank transfer at 120 V for 30 min in Tris-glycine buffer with 20% methanol. Membranes were blocked with Tris-buffered saline with Tween-20 containing 5% skim milk for 2 h at room temperature, then incubated overnight at 4°C with primary antibodies: anti-LC3B rabbit antibody (Sigma-Aldrich; lot: 046M4787V; 1:2000) and anti-β-actin rabbit antibody (CST; lot: 6; 1:2000). Afterward, membranes were incubated with horseradish peroxidase-conjugated goat anti-rabbit IgG secondary antibody for 1 hour, and immunoreactive bands were visualized using an enhanced chemiluminescence substrate (Boster; lot: 13E18B96). Densitometric analysis was performed with ImageJ software. Antibody specificity was confirmed by detecting single bands at ~14 kDa and ~16 kDa for LC3, and ~42 kDa for β-actin, aligning with previous research (Zhang et al., 2023). The LC3II/I ratio was calculated as (LC3II/β-actin) ÷ (LC3I/β-actin).

### Statistical analysis

Data were assessed for normality with the Shapiro–Wilk test and for homogeneity of variance with Levene’s test. One-way analysis of variance followed by Tukey’s multiple comparison test was conducted using SPSS 19.0 (SPSS Inc., Chicago, IL, USA). Data are presented as mean ± SE. Differences were regarded as not significant if p > 0.05, significant if p < 0.05, and highly significant if p < 0.01. Graphs were created with GraphPad Prism 9.2.0.

## RESULTS

### Infection dynamics of chicken cecal epithelial cells infected with *E. tenella in vitro*

**Figure 1:**
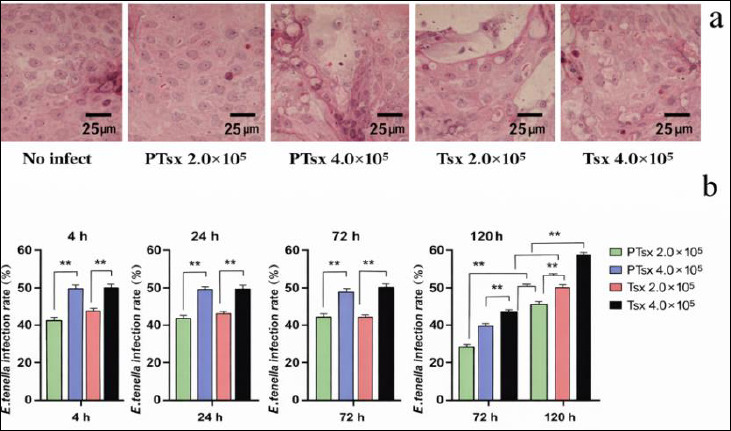
Infection of *Eimeria tenella* in chick embryo cecal epithelial cells *in vitro*. (a) Representative hematoxylin and eosin–stained micrograph of chicken cecal epithelial cells at 4 h after *E. tenella* infection (×400). (b) Quantitative analysis of infection rates at 4, 24, 72, and 120 h post-infection (n = 5). * indicates significant differences (p < 0.05), and ** indicates highly significant differences (p < 0.01).

No significant differences were observed in the infection rates of cecal epithelial cells between groups inoculated with the same dose of PTsx or Tsx at 4, 24, and 72 h post-inoculation (p > 0.05). However, at 120 h, infection rates in the PTsx groups were significantly lower than those in the Tsx groups (p < 0.01). In addition, infection rates in the high-dose groups (4.0 × 10^5^/well) were significantly higher than those in the low-dose groups (2.0 × 10^5^/well) at corresponding time points following *E. tenella* inoculation (p < 0.01) (Figure 1; Table 1).

**Table 1:** *In vitro* infection and autophagy responses of chicken cecal epithelial cells following infection with virulent (Tsx) and precocious (PTsx) *Eimeria tenella* strains at different time points.

Group	Infection Rate (%)	*Beclin-1* mRNA (Fold of control)	LC3II (dots/cell)	LC3II/I ratio
At 4 h after infection with *E. tenella*				
Control	0.0 ± 0.0	1.0036±0.0242	1.4±0.2449	0.7094±0.0162
PTsx low-dose	26.2 ± 0.86	0.9952±0.0224	3.8±0.3741	0.6324±0.0166
PTsx high-dose	41.6 ± 0.9	1.4405±0.0118	10.8±0.3741	0.633±0.0402
Tsx low-dose	27.0 ± 1.1	1.7098±0.0526	16.6±0.5099	0.7438±0.017
Tsx high-dose	42.4 ± 0.9	2.1204±0.0275	20.6±0.4	0.8303±0.0492
At 24 h after infection with *E. tenella*				
Control	0.0 ± 0.0	0.999±0.0227	2.4±0.2449	0.5564±0.0147
PTsx low-dose	23.8 ± 0.37	1.0574±0.0182	8.2±0.4	0.5583±0.0241
PTsx high-dose	39.2 ± 0.58	1.1981±0.0222	13.8±0.6	0.6477±0.0255
Tsx low-dose	23.8 ± 0.58	1.7083±0.0139	20.2±0.4	0.8565±0.0731
Tsx high-dose	39.6 ± 0.51	2.1507±0.0534	23.8±0.6	1.0822±0.0549
At 72 h after infection with *E. tenella*				
Control	0.0 ± 0.0	0.99887±0.0174	3.8±0.3741	0.5539±0.0205
PTsx low-dose	19.4 ± 0.51	1.0426±0.0123	10.8±0.4	0.5161±0.0201
PTsx high-dose	28.4 ± 0.51	1.3221±0.0189	16.8±0.4	0.5299±0.0068
Tsx low-dose	19.6 ± 0.51	1.9424±0.0258	24.8±0.6	0.8538±0.027
Tsx high-dose	29.4 ± 0.51	2.489±0.0411	27.8±0.4	0.9133±0.0204
At 120 h after infection with *E. tenella*				
Control	0.0 ± 0.0	0.9999±0.0153	4.4±0.4	0.5706±0.0116
PTsx low-dose	5.0 ± 0.31	1.072±0.0212	11.4±0.5099	0.6327±0.0249
PTsx high-dose	7.6 ± 0.24	1.4861±0.0465	17.2±0.3741	0.8703±0.0529
Tsx low-dose	11.4 ± 0.24	2.0002±0.034	26.2±0.3741	0.9383±0.0483
Tsx high-dose	18.2 ± 0.37	3.0895±0.0412	31.4±0.4	1.6947±0.0505

Tsx = virulent *Eimeria tenella* Shanxi strain, PTsx = precocious Eimeria tenella Shanxi strain, LC3 = microtubule-associated protein 1 light chain 3, Statistical significance was determined by one-way analysis of variance followed by Tukey's post hoc test; p < 0.05 was considered significant, p < 0.01 highly significant.

### Accumulation of LC3II in host cells infected with *E. tenella in vitro*

**Figure 2:**
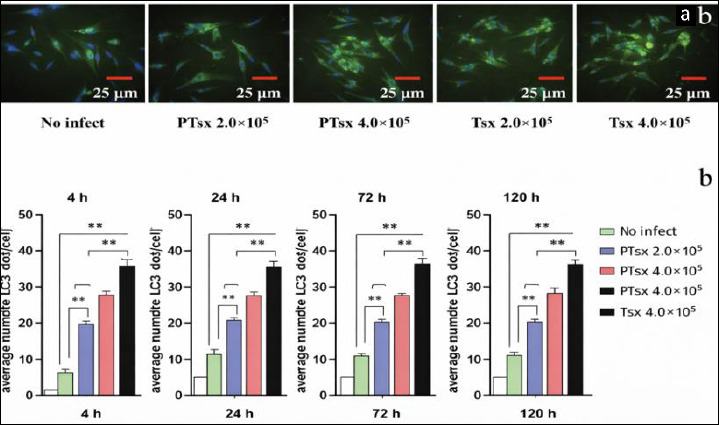
*Eimeria tenella* infection increases punctate LC3II accumulation in host cells. (a) Representative immunofluo-rescence micrograph showing punctate LC3II signals in chicken cecal epithelial cells at 4 h after *E. tenella* infection (×400). (b) Quantitative analysis of the mean number of LC3II puncta per cell at 4, 24, 72, and 120 h post-infection (n = 5). *indicates significant differences (p < 0.05), and ** indicates highly significant differences (p < 0.01).

The number of punctate LC3II dots in host cells infected with PTsx or Tsx at both low (2.0 × 10^5^/well) and high (4.0 × 10^5^/well) doses was significantly higher than that in the non-inoculated control group at 4–120 h post-infection (p < 0.01). At equivalent doses, PTsx-inoculated cells exhibited significantly fewer LC3II puncta than Tsx-inoculated cells (p < 0.01). Moreover, within each strain, the high-dose groups showed significantly greater LC3II puncta accumulation compared with the corresponding low-dose groups (p < 0.01) (Figure 2; Table 1).

### Relative expression of *Beclin-1* mRNA in *E. tenella*–infected host cells *in vitro*

**Figure 3:**
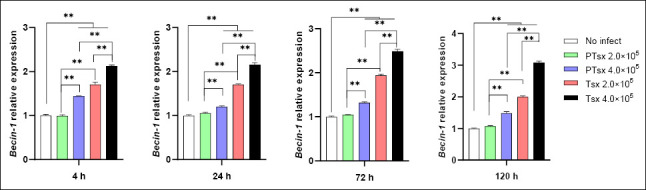
Relative *Beclin-1* mRNA expression in *Eimeria tenella*–infected host cells *in vitro*. *Beclin-1* mRNA levels were quantified in chicken cecal epithelial cells at different time points following *E. tenella* infection. * indicates significant differences (p < 0.05), and ** indicates highly significant differences (p < 0.01). Biological replicates: *in vitro* (n = 5 independent wells per group per time point); technical replicates: quantitative real-time polymerase chain reaction (three replicates per sample).

*Beclin-1* mRNA expression was significantly upregulated in the PTsx high-dose group (4.0 × 10^5^/well) and in both Tsx-inoculated groups (2.0 × 10^5^ and 4.0 × 10^5^/well) compared with the non-inoculated control at 4–120 h post-infection (p < 0.01). At the same inoculation dose, *Beclin-1* mRNA expression was significantly lower in PTsx-infected cells than in Tsx-infected cells (p < 0.01). Furthermore, *Beclin-1* mRNA levels were significantly higher in high-dose groups than in low-dose groups for the same strain (p < 0.01) (Figure 3; Table 1).

### LC3 expression in host cells infected with *E. tenella in vitro*

**Figure 4:**
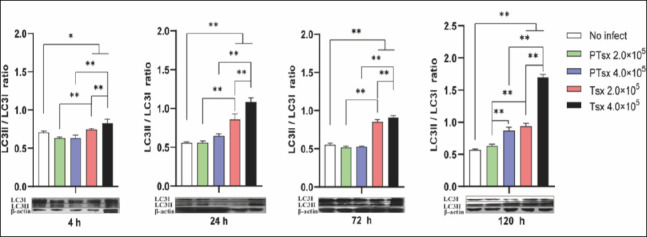
LC3II/LC3I protein ratio in *Eimeria tenella*–infected host cells *in vitro*. The LC3II/LC3I ratio was determined in chicken cecal epithelial cells at different time points following *E. tenella* infection to assess autophagy activation. * indicates significant differences (p < 0.05), and ** indicates highly significant differences (p < 0.01). Biological replicates: *in vitro* (n = 5 independent wells per group per time point); technical replicates: Western blot analysis (two replicates per sample).

Western blot analysis demonstrated that the LC3II/I ratio in Tsx-inoculated groups was markedly higher than in both non-inoculated and PTsx-inoculated groups between 4 and 72 h after inoculation (p < 0.05 or p < 0.01). No significant difference was observed between PTsx-inoculated and non-inoculated groups during this period (p > 0.05). At 120 h, the LC3II/I ratio was significantly elevated in the high-dose PTsx group (4.0 × 10^5^/well) and in both Tsx-inoculated groups compared to controls (p < 0.05). At the same doses, PTsx-inoculated cells had significantly lower LC3II/I ratios than Tsx-inoculated cells (p < 0.05 or p < 0.01), and high-dose groups presented higher ratios than low-dose groups for the same strain (p < 0.05 or p < 0.01) (Figure 4; Table 1).

### Relative expression of *Beclin-1* mRNA in *E. tenella*–infected cecal tissues *in vivo*

**Figure 5:**
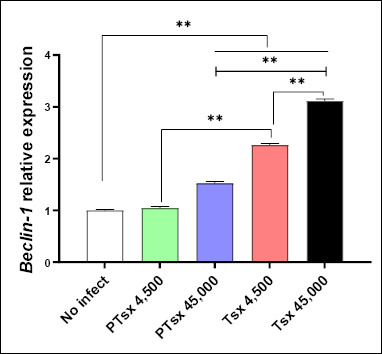
Relative *Beclin-1* mRNA expression in the cecum following *Eimeria tenella* infection *in vivo*. *Beclin-1* mRNA levels were measured in cecal tissues of chickens on day 5 after inoculation with *E. tenella*. * indicates significant differences (p < 0.05), and ** indicates highly significant differences (p < 0.01). Biological replicates: *in vivo* (n = 10 chickens per group); technical replicates: quantitative real-time polymerase chain reaction (three replicates per sample).

On day 5 post-inoculation, *Beclin-1* mRNA expression in the cecal tissues of chickens inoculated with PTsx at 45,000 oocysts/chicken and with Tsx at either 4,500 or 45,000 oocysts/chicken was significantly higher than that in the non-inoculated control group (p < 0.01). *Beclin-1* mRNA levels in PTsx-inoculated chickens were significantly lower than those in Tsx-inoculated chickens at corresponding doses (p < 0.01). Additionally, *Beclin-1* mRNA expression in the high-dose groups (45,000 oocysts/chicken) was significantly greater than that in the low-dose groups (4,500 oocysts/chicken) for the same strain (p < 0.01) (Figure 5; Table 2).

**Table 2:** Autophagy-related responses in chicken cecal tissues on day 5 after oral inoculation with virulent (Tsx) or precocious (PTsx) *Eimeria tenella*.

Group	*Beclin-1* mRNA(Fold of Control)	LC3II/I ratio(dots/cell)
Control	0.9985±0.0198	2.9239±0.1828
PTsx low-dose	1.044±0.0302	2.5307±0.0983
PTsx high-dose	1.5217±0.0362	3.9726±0.0706
Tsx low-dose	2.2616±0.0286	4.3701±0.0483
Tsx high-dose	3.1109±0.0393	9.1998±0.5531

Tsx = virulent *Eimeria tenella* Shanxi strain, PTsx = precocious Eimeria tenella Shanxi strain, LC3 = microtubule-associated protein 1 light chain 3. Statistical analysis was performed using one-way analysis of variance with Tukey's multiple comparison test; p < 0.05 indicates statistical significance.

### LC3 expression in the cecum of *E. tenella*–infected chickens *in vivo*

**Figure 6:**
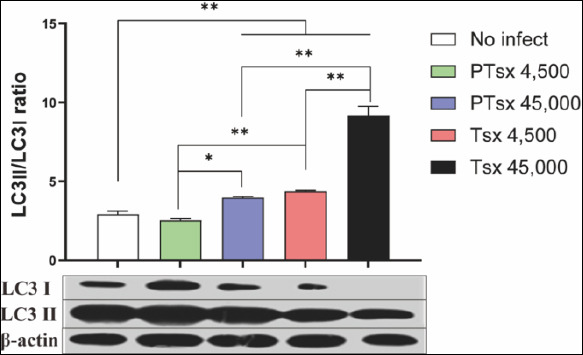
LC3II/LC3I protein ratio in cecal tissues following *Eimeria tenella* infection *in vivo*. The LC3II/LC3I ratio was determined in chicken cecal tissues on day 5 after inoculation with *E. tenella* to evaluate autophagy activation. * indicates significant differences (p < 0.05), and ** indicates highly significant differences (p < 0.01). Biological replicates: *in vivo* (n = 10 chickens per group); technical replicates: Western blot analysis (two replicates per sample).

The LC3II/I ratio in cecal tissues from the PTsx high-dose group and from both Tsx-inoculated groups was significantly higher than that in the non-inoculated group on day 5 post-infection (p < 0.05). At equivalent doses, PTsx-inoculated groups showed significantly lower LC3II/I ratios than Tsx-inoculated groups (p < 0.05 or p < 0.01). Furthermore, LC3II/I ratios were significantly higher in the 45,000 oocysts/chicken groups than in the 4,500 oocysts/chicken groups for the same strain (p < 0.05) (Figure 6; Table 2).

### Comparison of autophagy responses *in vitro* and *in vivo*

Autophagy markers, including *Beclin-1* mRNA expression and LC3II/I ratios, showed consistent strain- and dose-dependent trends across time points *in vitro* (4–120 h) and *in vivo* (day 5 post-inoculation), although autophagy activation peaked earlier *in vivo*. The LC3II/I ratio was approximately 4.0–5.4-fold higher *in vivo* than *in vitro*, particularly in high-dose Tsx groups, likely reflecting contributions from inflammatory responses and microbial signals in the intestinal microenvironment. Basal autophagy levels were also higher in cecal tissues *in vivo* (LC3II/I = 2.9239 ± 0.1828) than in cultured epithelial cells *in vitro* (0.5706 ± 0.0116). Notably, PTsx-induced autophagy was comparatively mild *in vivo* and was associated with reduced intestinal damage, supporting the relative safety of PTsx as a live attenuated vaccine candidate (Tables 1–3).

**Table 3:** Comparative analysis of autophagy markers between *in vitro* and *in vivo* *Eimeria tenella* infection models.

Group	*Beclin-1* mRNA (Fold of Control)		LC3II/I ratio (dots/cell)	
	
	*in vitro*	*in vivo*	*in vitro*	*in vivo*
Control	0.9999±0.0153	0.9985±0.0198	0.5706±0.0116	2.9239±0.1828
PTsx low-dose	1.072±0.0212	1.044±0.0302	0.6327±0.0249	2.5307±0.0983
PTsx high-dose	1.4861±0.0465	1.5217±0.0362	0.8703±0.0529	3.9726±0.0706
Tsx low-dose	2.0002±0.034	2.2616±0.0286	0.9383±0.0483	4.3701±0.0483
Tsx high-dose	3.0895±0.0412	3.1109±0.0393	1.6947±0.0505	9.1998±0.5531

Tsx = virulent *Eimeria tenella* Shanxi strain, PTsx = precocious Eimeria tenella Shanxi strain, LC3 = microtubule-associated protein 1 light chain 3. Data are expressed as mean ± standard error.

## DISCUSSION

### Autophagy as a host cellular process during protozoan infection

Autophagy is a conserved cellular process in eukaryotic cells in which double-membrane vesicles sequester excess or damaged organelles and deliver them to lysosomes or vacuoles for degradation, thereby facilitating cellular recycling and removal of harmful components. In addition to apoptosis and necrosis, autophagy is recognized as a distinct form of programmed cell death [18]. Several intracellular protozoa, including *Toxoplasma gondii* and *Cryptosporidium parvum*, are known to induce host cell autophagy during infection [19, 20]. Previous work by our research group demonstrated that large numbers of double-membrane autophagosomes were formed in chick embryo cecal epithelial cells 72 h after *E. tenella* infection, indicating that *E. tenella* can actively induce autophagosome formation in host cells. In parallel, the transcriptional and protein expression levels of autophagy-related factors, including *autophagy-related* gene (*ATG*)*5*, *Beclin-1*, and *LC3*, were significantly upregulated, confirming that *E. tenella* promotes host cell autophagy [14].

### Strain- and dose-dependent induction of autophagy by *E. tenella in vitro*

The virulent *E. tenella* strain Tsx strongly activates autophagy and promotes autophagosome–lysosome fusion. Using drugs that modulate autophagy, such as the inducer rapamycin (RAPA) and the inhibitor 3-methyladenine (3-MA), which target the mTOR and Phosphatidylinositol 3-kinase pathways, respectively, can alter host cell autophagy levels, thereby decreasing or increasing *E. tenella* infection rates. Additionally, chloroquine significantly elevated LC3B-II and p62 levels in *host cells* infected with E. tenella at 72 h post-infection [14]. In this study, the expression levels of *Beclin-1* mRNA, the number of punctate LC3II signals, and the LC3II/I ratio were notably higher in the high-dose precocious strain group and all virulent strain–inoculated groups compared to controls following 4–120 h of i*n vitro* infection. At the same inoculation doses, these autophagy markers were consistently higher in virulent strain groups than in precocious strain groups and higher in high-dose groups than in low-dose groups. These results suggest that the virulent *E. tenella* strain induces stronger autophagy in host cells than the precocious strain at the same dose, and that autophagy induction correlates positively with infection dose.

### Autophagy as a double-edged sword in host–parasite interactions

Evidence shows that autophagy is essential for host defense against intracellular pathogens, but many pathogens have developed ways to evade or harness autophagy to survive and replicate within host cells [21, 22]. For instance, *Plasmodium* infection triggers autophagy in liver cells, yet the parasite can grow inside autophagic vesicles [23]. Enhancing host autophagy through drugs increases *Plasmodium* growth and infection rates, while blocking autophagy reduces parasite development and infection [24]. Similarly, *Toxoplasma gondii* avoids immune-related autophagy by creating parasitophorous vacuoles and also uses nutrients derived from autophagy to sustain persistent infection [25]. These findings align with our current results showing that the PTsx strain induces weaker autophagy activation compared to the virulent Tsx strain at the same dose, resulting in only mild and temporary activation of the autophagic pathway.

### Potential molecular mechanisms underlying differential autophagy induction

The differential induction of autophagy by Tsx and PTsx may involve multiple signaling pathways, such as mTOR/AMPK (AMP-activated protein kinase) and Ca^2+^-dependent signaling. Tsx’s greater virulence and ability to proliferate likely cause more nutrient stress in host cells, leading to the activation of AMPK and suppression of mTOR, which promotes autophagy [21]. Conversely, PTsx has roughly 40% lower reproductive capacity and shows reduced Ca^2+^ dysregulation [15], potentially diminishing endoplasmic reticulum stress and reactive oxygen species, thus limiting autophagy activation. Additionally, rhoptry proteins (ROPs), key virulence factors, are expressed at lower levels in attenuated strains, possibly affecting autophagy via changes in inflammatory signaling and nutrient requirements.

### Temporal infection dynamics and host cell fate regulation

No notable differences in host cell infection rates were observed between precocious and virulent strains from 4 to 72 h after *in vitro* inoculation. However, at 120 h, the virulent strain group showed significantly higher infection rates. This may be due to incomplete schizogony of second-generation merozoites in the precocious strain, leading to fewer progeny, lower nutrient needs, and less host cell damage. These phenotypic differences could limit the precocious strain’s ability to induce long-term host cell autophagy, though further validation is needed. While autophagy acts as a cellular defense mechanism, sustained hyperactivation, ike that seen during Tsx infection from 4 to 120 h, may cause negative effects such as nutrient imbalance, cell damage, and immune dysregulation. In early *E. tenella* infection, the parasite seems to promote host cell autophagy while blocking apoptosis, ensuring nutrient supply and extending host cell survival to support its development.

### Comparison of autophagy responses *in vivo* and *in vitro*

On day 5 after inoculation *in vivo*, *Beclin-1* mRNA levels and LC3II/I ratios increased in the high-dose precocious strain group and all virulent strain groups compared to non-inoculated controls, except for the low-dose precocious group. At similar doses, autophagy markers were consistently higher in chickens infected with virulent strains than in those with precocious strains, and higher in high-dose groups than in low-dose groups. Notably, *Beclin-1* mRNA levels showed similar activation levels *in vivo* and *in vitro* (e.g., Tsx high-dose group: 3.1109 ± 0.0393 *in vivo* vs. 3.0895 ± 0.0412 *in vitro*), indicating a conserved, strain- and dose-dependent regulation of autophagy initiation. Conversely, LC3II/I ratios were significantly higher *in vivo*, with increases of 4.0-fold in the PTsx low-dose group and 5.4-fold in the Tsx high-dose group compared to *in vitro* measurements. This amplification probably reflects the impact of the intestinal microenvironment, including immune cells, microbiota-derived metabolites like lipopolysaccharides and short-chain fatty acids, as well as cytokines such as IFN-γ and IL-1β.

### Implications for vaccine safety and future research directions

The relatively mild induction of autophagy by PTsx likely helps reduce nutrient depletion in host cells and prevents intestinal damage. This offers a mechanistic explanation for the lesser growth suppression seen in vaccinated chickens compared to those infected with Tsx [8, 9, 11]. The dose-dependent autophagy responses observed support optimizing PTsx vaccination doses (for example, 4,500 oocysts per chicken) to achieve a balance between immune response and safety, while also minimizing adverse effects related to autophagy. Although the study clearly links host cell autophagy, *E. tenella* strain virulence, and infection dose, it is still unknown whether PTsx’s limited ability to induce autophagy reflects co-evolution between host and parasite. PTsx might improve host cell adaptability by restricting second-generation schizont development and reducing autophagic stress, thus aiding parasite survival. This research primarily examined autophagy marker expression and did not measure autophagic flux with lysosomal inhibitors like chloroquine or bafilomycin A1. Future research incorporating flux assays and exploring interactions between autophagy, innate immune responses, and gut microbiota will be crucial to fully understand the mechanisms behind *Eimeria*–host interactions.

## CONCLUSION

This study showed that infection with *E. tenella* triggers host cell autophagy in a strain- and dose-dependent manner, both *in vitro* and *in vivo*. The virulent Tsx strain consistently caused stronger autophagy activation than the attenuated PTsx strain, as indicated by significantly higher *Beclin-1* mRNA levels, increased LC3II puncta, and higher LC3II/I ratios. Autophagy responses grew with the infection dose for both strains. Although *Beclin-1* activation was similar in amplitude *in vitro* and *in vivo*, the LC3II/I ratios were notably higher *in vivo*, especially in high-dose Tsx groups, emphasizing the influence of the intestinal microenvironment.

The milder autophagy induction observed with the PTsx strain explains its reduced intestinal damage and lesser impact on growth performance compared with the virulent Tsx strain. These findings provide a mechanistic basis for optimizing live attenuated coccidiosis vaccines by selecting strains and doses that balance immunogenicity with minimal autophagy-associated tissue injury. Specifically, lower PTsx doses may be sufficient to induce protective immunity while limiting adverse effects, supporting safer vaccination strategies in poultry production.

A key strength of this study is its integrated comparison of virulent and precocious *E. tenella* strains using both *in vitro* and *in vivo* models. Employing multiple autophagy markers (*Beclin-1* and LC3) allowed for a thorough evaluation of autophagy initiation and autophagosome formation. The consistent pattern of strain- and dose-dependent trends across different experimental systems enhances the biological significance and translational potential of the results.

This study primarily measured autophagy via marker expression and did not directly assess autophagic flux using lysosomal inhibitors such as chloroquine or bafilomycin A1. Furthermore, the *in vitro* model lacked immune cells and intestinal microbiota, which restricts its ability to fully replicate the complex host–parasite interactions seen *in vivo*. These limitations might partly account for the differences in autophagy responses observed between *in vitro* and *in vivo* experiments.

Future investigations should incorporate autophagic flux assays to distinguish between increased autophagosome formation and impaired degradation. Studies integrating immune signaling pathways, microbiota composition, and metabolic profiling will be critical to clarify how autophagy intersects with intestinal immunity during *E. tenella* infection. Moreover, exploring the role of parasite virulence factors in modulating host autophagy may inform rational design of next-generation attenuated vaccines.

This study demonstrates that host cell autophagy is a key process connecting *E. tenella* virulence, infection dose, and intestinal damage. The weakened PTsx strain triggers controlled autophagy that reduces host harm while maintaining immune response, highlighting its potential as a live vaccine candidate. These results enhance our understanding of *Eimeria*–host dynamics and offer a foundation for developing safer and more effective coccidiosis management strategies.

## DATA AVAILABILITY

All the generated data are included in the manuscript.

## AUTHORS’ CONTRIBUTIONS

Li Zhang: Carried out most of the experiments and wrote the manuscript. Ying-ying Chen, Hong-hui Zhang and Xiao-zhen Cui: Helped with the experiment. Ming-Xue Zheng and Long-long Zheng: Revised the manuscript and the experiment design. All the authors read and approved the final version of the manuscript.
